# Distinct roles for IκB kinases alpha and beta in regulating pulmonary endothelial angiogenic function during late lung development

**DOI:** 10.1111/jcmm.13741

**Published:** 2018-07-11

**Authors:** Cristiana Iosef, Min Liu, Lihua Ying, Shailaja P. Rao, Katherine R. Concepcion, Westin K. Chan, Andrew Oman, Cristina M. Alvira

**Affiliations:** ^1^ Department of Pharmacology Faculty of Medicine University of Nevada Reno Reno NV USA; ^2^ Department of Pediatrics Center for Excellence in Pulmonary Biology Stanford University School of Medicine Stanford CA USA

**Keywords:** alveolarization, angiogenesis, endothelial cells, lung development, nuclear factor kappa‐B

## Abstract

Pulmonary angiogenesis is essential for alveolarization, the final stage of lung development that markedly increases gas exchange surface area. We recently demonstrated that activation of the nuclear factor kappa‐B (NFκB) pathway promotes pulmonary angiogenesis during alveolarization. However, the mechanisms activating NFκB in the pulmonary endothelium, and its downstream targets are not known. In this study, we sought to delineate the specific roles for the NFκB activating kinases, IKKα and IKKβ, in promoting developmental pulmonary angiogenesis. Microarray analysis of primary pulmonary endothelial cells (PECs) after silencing IKKα or IKKβ demonstrated that the 2 kinases regulate unique panels of genes, with few shared targets. Although silencing IKKα induced mild impairments in angiogenic function, silencing IKKβ induced more severe angiogenic defects and decreased vascular cell adhesion molecule expression, an IKKβ regulated target essential for both PEC adhesion and migration. Taken together, these data show that IKKα and IKKβ regulate unique genes in PEC, resulting in differential effects on angiogenesis upon inhibition, and identify IKKβ as the predominant regulator of pulmonary angiogenesis during alveolarization. These data suggest that therapeutic strategies to specifically enhance IKKβ activity in the pulmonary endothelium may hold promise to enhance lung growth in diseases marked by altered alveolarization.

## 1 INTRODUCTION

Pulmonary vasculature growth by angiogenesis is essential for alveolarization, the final stage of lung development that occurs after term birth, and markedly increases the gas exchange surface area of the lung.^1^ Angiogenic factors normally increase during late lung development, but are suppressed by injuries that disrupt alveolarization.^2^ Pulmonary angiogenesis and alveolarization are impaired in premature infants dying from bronchopulmonary dysplasia (BPD),^3^ a chronic lung disease that is the most common complication of premature birth. Although the link between angiogenesis and alveolarization is clear, the molecular pathways that orchestrate pulmonary angiogenesis during alveolarization remain to be elucidated.

Accumulating evidence indicates that the nuclear factor kappa B (NFκB) signalling pathway plays a key role in both physiologic and pathologic angiogenesis. The NFκB family of transcription factors consists of 5 members each able to bind DNA, and dimerize with other family members.^4^ Canonical activation of NFκB occurs by phosphorylation and degradation of IκBs by the IκB kinases, IKKα and IKKβ, resulting in rapid translocation of active NFκB complexes into the nucleus to regulate critical genes in cell functions.^5^ NFκB enhances angiogenic sprouting during wound healing, and promotes tumour angiogenesis by increasing angiogenic molecules such as vascular endothelial growth factor (VEGF).^6^, ^7^ Further, we recently showed that NFκB is constitutively active in the neonatal pulmonary endothelium but quiescent in the adult tissue.^8^ Administration of a pharmacologic inhibitor of IKKα and IKKβ disrupts alveolarization in neonatal mice, and impairs survival, proliferation and in vitro angiogenesis of primary pulmonary endothelial cells (PECs) isolated from the early alveolar lung.^8^

However, the mechanisms allowing temporal‐specific activation of NFκB in the pulmonary circulation during alveolarization are not known. IKKα and IKKβ have structurally similar catalytic subunits, and both kinases are able to phosphorylate IκB and induce canonical activation of NFκB dimers.^9^ However, IKKα and IKKβ also possess NFκB‐independent functions, allowing unique role for the 2 kinases, and likely accounting for the contradictory results frequently obtained in experimental studies targeting IKKα and IKKβ individually. For example, IKKα is increased in the vasculature of lung adenocarcinomas, and over‐expression of IKKα, but not IKKβ, in HUVECs promotes in vitro angiogenesis.^10^ In contrast, homozygous deletion of IKKβ in endothelial cells disrupts placental vascularization and impairs EC survival and migration.^11^ Taken together, these data suggested that IKKα and IKKβ may direct independent functions in the vasculature, with each kinase serving as the predominant regulator of angiogenesis depending on the tissue type and/or stage of development.

Therefore, in this study, we sought to determine the specific roles for IKKα and IKKβ in the pulmonary vasculature during postnatal lung development. We performed microarray analysis of gene expression in neonatal PECs after silencing either IKKα or IKKβ, and found that the 2 kinases regulate unique panels of genes in the pulmonary vasculature. The loss of either kinase resulted in distinct impairments in physiologic functions essential for angiogenesis including, proliferation, survival, adhesion, migration and cytoskeletal remodelling. Although the number of dysregulated genes was higher in response to silencing IKKα, the functional effects on angiogenic function were substantially greater with loss of IKKβ. Taken together, these data suggest that IKKα and IKKβ regulate distinct but complementary patterns of genes important for angiogenic function in the pulmonary circulation and identify IKKβ as the predominant regulator of angiogenesis in the pulmonary endothelium during postnatal lung development.

## 2 METHODS

### 2.1 PEC isolation

Neonatal mouse (6 day old, C57BL/6) lungs were digested with collagenase IA (0.5 mg/mL) for 30 minutes at 37°C, and resulted cells were incubated with anti‐CD31‐coated magnetic beads (Dynabeads, Invitrogen). PECs were isolated by magnetic separation and cultivated as previously described.^8^ A description of the characterization of these cells is provided in our previously published manuscript by Iosef et al^8^, where we showed that PEC isolated by this method were found to be exclusively CD45(−) and greater than 95% CD31(+)/CD102(+) by flow cytometry.

### 2.2 RNA interference

Pulmonary endothelial cells were transfected using either IKKα or IKKβ On‐Target Plus SMART pool siRNA or non‐targeting siRNA as a control (Dharmacon; Thermo Fisher Scientific) and Lipofectamine 2000 (Invitrogen) for 6 hours. RNA was isolated at 36 hours using the RNeasy kit (Qiagen, Valencia, CA, USA) for qPCR, and whole cell protein lysate was extracted at 42 hours for Western blot. In separate experiments, additional groups of siRNA‐treated cells were used at 40 hours post‐transfection for functional assays.

### 2.3 Microarray

Gene expression patterns were assessed using GeneChip^®^ PrimeView™ Mouse Gene Expression Array (Affymetrix) according to the manufacturer's protocols. Single‐stranded cDNA was synthesized from total RNA obtained from PEC transfected with NTC, anti‐IKKα or anti‐IKKβ siRNA and hybridized to gene arrays. Differentially expressed genes were determined by Significance Analysis of Microarray (SAM) method and by R software. Drafting principles were applied by fold change ≥2 or ≤0.5, and *q*‐value ≤5%. Further analysis was then performed with iPathwaysGuide from Advaita Bioinformatics (Plymouth, MI, USA) to assess gene ontology and enriched functional pathways with pathway topologies, based on Kyoto Encyclopedia of Genes and Genomes (KEGG) Ontology Based Annotation System (KOBAS 2.0) database.^12^ Comparisons between groups and Meta‐Analysis were performed, revealing common and differential gene expression in the 3 groups of PEC.

### 2.4 Quantitative real‐time PCR (qRT‐PCR)

Total RNA was isolated from PEC and whole mouse lung using the RNeasy kit (Qiagen). RNA (2 μg) was reverse‐transcribed using Superscript III (Invitrogen), and qPCR was performed using TaqMan primers (Applied Biosystems) as described previously.^13^ Quantification of target gene expression was performed using the standard curve method and normalizing to the expression of either 18s or β2M which were both stable in our transfected cells through experiments performed over several years.

### 2.5 Proliferation assays

At 40 hours post‐transfection, 6 × 10^3^ cells were plated into wells of 96‐well plates and starved (0.2% FBS) for 8‐12 hours. The starvation media was then removed and bromodeoxyuridine (BrdU) added to each of the following conditions: complete EGM (Endothelial Growth Medium) + 5% FBS; EGM stripped by all growth factors + 0.2% FBS; EGM stripped by all growth factors + 0.2% FBS + IGF‐1, EGF, VEGF, or FGF2 (all at 50 ng/mL). Incorporation of BrdU was measured by ELISA at 2‐24 hours per the manufacturer's protocol (Roche Diagnostics).

### 2.6 Apoptosis assays

At 24 hours post‐transfection, cells were transferred to 96‐well plates (1 × 10^4^ cells/well) and after reaching 80%‐90% confluence, the media was exchanged for starvation media (FBS 0.2% without additional growth factors). Separate groups of cells were exposed to TNF‐α (20 ng/mL) to induce apoptosis for 4, 8 and 24 hours. Apoptosis was measured by determining the amount of active caspase 3/7 using a Luciferase‐Glo system (Promega). The assays were carried out per manufacturer's protocol, and the luciferase reactions read using a Berthold LG&G Lumat LG luminometer as performed previously.^8^

### 2.7 Cell adhesion

At 48 hours post‐siRNA transfection, PECs were plated at a density of 2 × 10^4^ cells/well, in either gelatine (0.2%) coated culture slides or glass chamber slides pre‐coated with different extracellular matrix proteins (BD variety pack). At 1 hour post‐plating, non‐adherent cells were removed, and adherent cells fixed and F‐actin stained with Phalloidine‐TRITC (ThermoFisher Scientific) and Hoechst reagent. Specimens were imaged with a Leica DM5500B Upright Microscope and Photometrics CoolSNAP HQ2 CCD camera, and Metamorph Image Analysis software was used to manually count the number of adherent cells in serial images of random microscopic fields of each specimen.

### 2.8 Western blot

Whole cell lysates were obtained from PEC using a lysis buffer containing 10 mmol/L Tris–HCl and 1% SDS, heated to 100°C. Protein lysates were subjected to SDS‐PAGE. Membranes were incubated with primary antibodies to detect Akt, pAKT, p38, ERK1/2 (Cell Signaling), tubulin (Abcam), IKKα and IKKβ (Cell Signaling) and the appropriate HRP‐conjugated secondary antibody, followed by enhanced chemiluminescence detection (GE Life Sciences).

### 2.9 Wound healing assay

At 24 hours post‐siRNA transfection, PECs were plated on BD‐Falcon culture slides coated with 0.2% gelatine. Once confluent, the monolayer was wounded with a fine pipette‐tip creating a wound along the diagonal axis of the well. The wound was permitted to heal by PEC migration for the next 24 hours. Phase contrast images were captured before and after the healing process using a Nikon Eclipse inverted microscope with NIS Elements software. The wound closure area was analysed using Image‐J software.

### 2.10 F‐Actin cytochemistry

NTC‐, IKKα‐ and IKKβ siRNA‐treated neonatal PECs were plated on glass coverslips 24 hours after transfection and starved for 12 hours. Starvation media (SM) was replaced with either new SM (FBS 0.2%, complete EGM (FBS 5% with growth factors), or SM with VEGF (50 ng/mL). Fixed cells were rehydrated, permeabilized and non‐specific binding diminished using Sea Block Blocking Solution (Thermo Scientific). Cells were then incubated with Phalloidin‐TRITC reagent (ThermoFisher Scientific) and counter‐stained for chromatin with Hoechst reagent (1 μg/mL) (Sigma‐Aldrich). Fluorescent images were captured using a Leica DM5500B Upright Microscope and Photometrics CoolSNAP HQ2 CCD camera, using HC Plan Apo 25‐mm objectives at ×40 magnification.

### 2.11 Tube formation assay

Pulmonary endothelial cells were transfected with NTC, IKKβ or VCAM siRNA as described above. At 48 hours after transfection, 3.5 × 10^4^ PECs were plated into 48‐well dishes coated with Reduced Growth Factor Basement Membrane Extract (Trevigen, Gaithersburg, MD) in the presence of VEGF‐A (50 ng/mL). For all experiments, representative images of tube networks were obtained at 8 hours, and the total tube length and branch points per 4X field was quantified in a blinded fashion by uploading images for evaluation using Wimasis Image Analysis software.

### 2.12 Statistics

All data are presented as means ± SE. Statistical differences between 2 groups were determined by Student's *t* test and between more than 2 groups by either one‐way or two‐way ANOVA, followed by Bonferroni's multiple‐comparison post hoc analysis. A *P* value of ≤.05 was considered statistically significant. All experiments have been performed with multiple biological and technical replicates, with specific replicate numbers detailed in the figure legends.

## 3 RESULTS

### 3.1 Silencing IKKα and IKKβ in PECs dysregulates unique panels of genes

**Figure 1:**
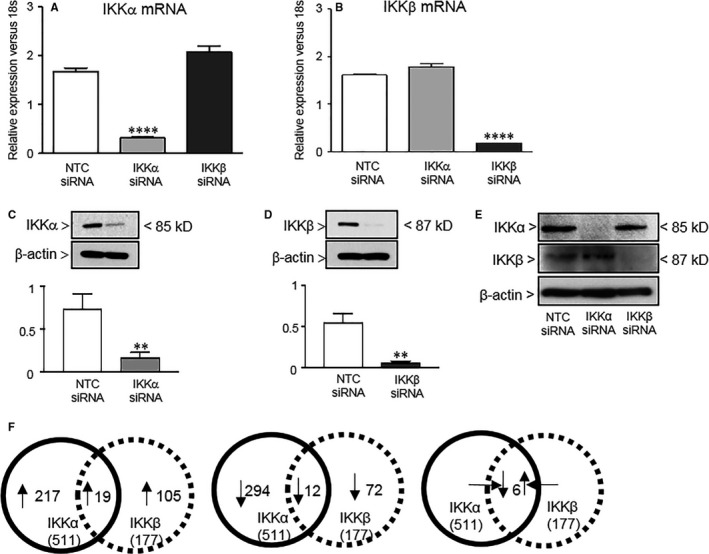
Silencing of IKKα and IKKβ in neonatal primary pulmonary endothelial cells dysregulates unique panels of genes. Quantitative PCR to detect (A) IKKα and (B) IKKβ gene expression relative to 18s in neonatal PEC treated with NTC, IKKα and IKKβ siRNA. Data shown are mean ± SEM with n = 12, and ****P < .0001 via one‐way ANOVA. Western blot to detect (C) IKKα and (D) IKKβ protein expression relative to β‐actin in neonatal PEC treated with NTC, IKKα and IKKβ siRNA. Data shown are mean ± SEM with n = 3, and **P = .0052 for NTC vs IKKα siRNA‐treated cells in (C) and **P = .0017 for NTC vs IKKβ siRNA treated cells in (D). (E) Representative Western blot to detect levels of IKKβ or IKKα compared to NTC when one or the other kinase were silenced by specific siRNAs. (F) Venn diagrams depicting shared and unique genes up‐ or down‐regulated in neonatal PEC after IKKα and IKKβ silencing. Loss of IKKα resulted in 511 dysregulated genes (217 up‐regulated and 294 down‐regulated), and loss of IKKβ resulted in 177 dysregulated genes (105 up‐regulated and 72 down‐regulated), with only 37 shared genes in the 2 groups. Of the 37 common genes, 19 were up‐regulated and 12 were down‐regulated by both IKKα and IKKβ siRNA. The remaining 6 shared targets were down‐regulated by IKKα siRNA but up‐regulated by IKKβ siRNA

To evaluate the distinct roles for IKKα vs IKKβ in neonatal PECs, we disrupted IKK signalling in a subunit‐specific manner by RNA interference. Transfection of PEC with siRNA to IKKα decreased IKKα mRNA levels by 82% (*P* < .0001 vs NTC siRNA, n = 12) without affecting IKKβ mRNA or protein levels (Figure 1A,E). Similarly, transfection of PEC with siRNA to IKKβ decreased IKKβ mRNA by 89% (*P* < .0001 vs NTC siRNA, n = 12), without altering IKKα mRNA or protein levels (Figure 1B,E). Western blot confirmed a significant reduction in IKK protein after siRNA transfection (Figure 1C,D), with a 76% reduction in IKKα (0.75 ± 0.1 vs 0.18 ± 0.03) and 87% reduction in IKKβ (0.55 ± 0.06 vs 0.07 ± 0.01). Next, genes regulated by IKKα and IKKβ were determined using GeneChip^®^ PrimeView™ Mouse Gene Expression Array (Affymetrix), in PEC transfected with NTC, anti‐IKKα or anti‐IKKβ siRNA. Silencing IKKα in PEC dysregulated 511 genes (up‐regulating 217 and down‐regulating 274), while silencing of IKKβ dysregulated 177 genes (up‐regulating 105 and down‐regulating 72). Interestingly, the number of shared targets was particularly small (37 genes) (Figure 1F). Out of the 37 common genes, 19 were up‐regulated by both IKKα and IKKβ siRNA, 12 were down‐regulated by both IKKα and IKKβ siRNA and 6 were down‐regulated by silencing IKKα, but up‐regulated by silencing by IKKβ. We then performed functional clustering using the Unified Human Interactome database (UniHI 4), and pathway analyses using i‐Pathway‐Guide software, and identified enrichment of genes in processes relevant including: cell cycle, proliferation, survival, apoptosis, cell adhesion, cytoskeletal organization, and G‐protein coupled signalling, and especially to angiogenesis (Table 1). These analyses identified distinct gene panels regulated by the 2 IKK kinases in neonatal PECs. Numerous gene targets of interest were validated by RT‐qPCR and found to have fidelity with the microarray results, including *VCAM, SELP, SPP, F2RL1, FAS, PRKX, MCM7, RBB4* (data not shown).

**Table 1:** IKKα and IKKβ regulate distinct panels of angiogenic genes in neonatal primary pulmonary endothelial cells. Selected IKKα and IKKβ up‐ or down‐regulated genes (>2x) relevant to the main cellular functions of pulmonary endothelial cells and angiogenesis, including motility, cell proliferation and differentiation, survival, and death or turnover

	IKKα		IKKβ	
	Up‐regulated	Down‐regulated	Up‐regulated	Down‐regulated
**Motility**				
Actin rearrangements	MYLC2B	PAK1, PFN2, ITGB4, RAC3, WASF2	ARPC1B	RAC3, CAPZA‐2
Adherent junctions	—	SELP, RAC3,WASF2, VCAM	—	RAC3, VCAM, F2RL‐1/PAR2
Focal adhesion	IGF1, MYLC2B, FLRT3	PAK1, CCND1, AKT3, SPP1, ITGB4, RAC3	IGF1, CCND2, FLT1, IGFBP‐5	RAC3, TNIP‐1, FBLN1
Tight junctions	F11R, MYLC2B	CCND1, MICALL2	MMP5	CLDN‐1
Migration	—	MAPK12/11, AKT3, RAC3	IGF1, FLT1, IGFBP‐5	RAC3
**Multiplication**				
Cell cycle checkpoints	RBX1, E2F5, SKP2, CDC23	CCND1, E2F3	CCND2	CDC2A, MCM7, NCAPD2
Proliferation	GAB1, IGF1, MAP3K1	PLA1A, PAK1, AKT3, RAC3, IKBKG	IGF1, PLA2G12A, FLT1, FGFRL1	RAC3, IKBKG, RGS16
**Diffentiation**				
FOXO pathway	IGF1,SKP2	CCND1, MAPK12/11, AKT3	IGF1, KLF2, CCND2	—
Stem cell maintenance	IGF1, RARB	MAPK12/11, AKT3, WINT2	IGF1, WNT2	ZFP281, CDC2A
**Death & turnover**				
Apoptosis	FAS	AKT3, ATF4, IKBKG	IGFBP‐5	IKBKG, SNCA, THOC‐1, DIABLO, CASP4
p53 pathways	EI24, IGF1, FAS	CCND1	IGF1, CCND2	ARIH‐2
TGF beta pathways	RBX1, E2F5	BMP7	—	—
TNF alpha pathways	FAS	MAPK12/11, AKT3, ATF4, VCAM, CCL2	—	—
**Survival**				
Global factors	IGF1	CCND1, AKT3, GNB1, ITGB4, ATF4, IKBKG	IGF1, CCND2, FLT1	HSP90AB1, IKBKG

### 3.2 Silencing IKKβ impairs PEC proliferation in response to selected growth factors

**Figure 2:**
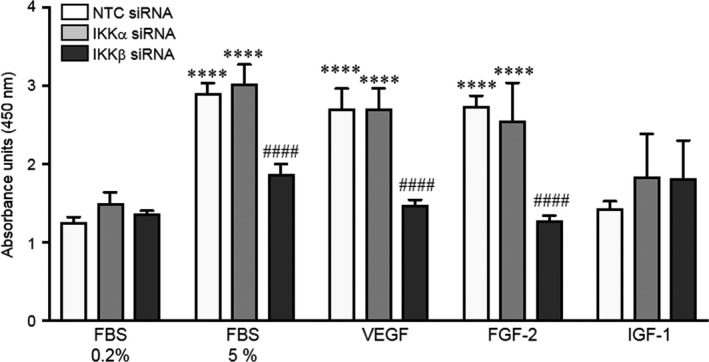
Silencing of IKKβ impairs PEC proliferation in response to select growth factors. BrdU incorporation assays were performed to measure proliferation in NTC siRNA‐treated, IKKα siRNA‐treated and IKKβ‐siRNA‐treated cells in response to starvation media containing FBS 0.2%, media containing FBS 5% or starvation media supplemented with either VEGF, FGF‐2 or IGF‐1. Data shown are mean ± SEM with n = 4, and ****P < .001 vs FBS 0.2%, and ^####^ *P* < .0001 vs NTC and IKKα siRNA via two‐way ANOVA

Analyses of the genes differentially regulated by the IKKs revealed multiple factors important in cell cycle checkpoints and proliferation which were altered by silencing IKKα, including cyclin D1 (CCND1),^14^ the transcription factor E2F3^15^ and Akt3.^16^ In contrast, fewer genes relevant to proliferation appeared dysregulated by IKKβ silencing but included up‐regulation of Flt1, a molecule able to antagonize VEGF‐mediated angiogenic function.^17^ To determine whether IKKα and IKKβ mediated regulation of these unique gene sets resulted in differential effects on proliferation, we performed BrdU incorporation assays on the siRNA transfected PEC in response to stimulation with complete endothelial growth media (EGM), or specific individual growth factors. This data are summarized in Figure 2. NTC siRNA‐treated cells proliferated robustly in response to EGM, VEGF or FGF‐2, but no proliferative response was observed with IGF‐1. IKKα siRNA‐treated PECs responded similarly to controls, demonstrating robust proliferation in response to complete EGM, VEGF and FGF‐2, but no proliferation in response to IGF‐1. In contrast, proliferation was broadly impaired in IKKβ siRNA‐treated cells, with no significant increase in proliferation in response to EGM, VEGF or FGF‐2. Taken together, these data suggest that IKKβ plays an indispensable role in promoting neonatal PEC proliferation.

### 3.3 Silencing IKKα or IKKβ impairs PEC survival

**Figure 3:**
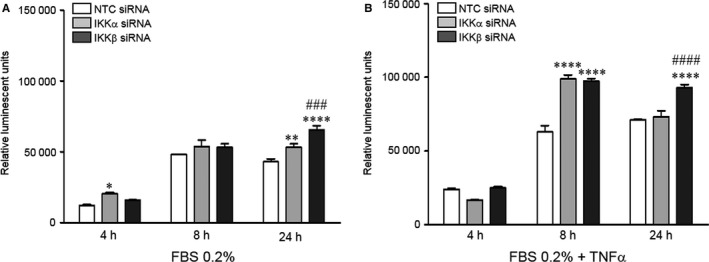
Silencing either IKKα or IKKβ impairs PEC survival. Apoptosis assays were performed to detect the amount of active caspase 3/7 in NTC‐ (white bars), IKKα (light grey bars) and IKKβ (dark grey bars) siRNA‐treated cells in response to (A) serum starvation and (B) TNF‐α stimulation at 4, 8 and 24 h. Data shown are mean ± SEM with n = 3. In both (A) and (B) for all siRNA treatment groups, apoptosis at 8 and 24 h was significantly greater than at 4 h, with *P < .05, **P < .01, and ****P < .0001 vs NTC siRNA at that time point; and ^###^ *P* < .001 and ^####^ *P* < .001 vs IKKα siRNA at that time point via two‐way ANOVA

The NFκB/IKK signalling pathway can have divergent effects on cell survival, inducing either pro‐ or anti‐apoptotic targets.^18^, ^19^ Our prior work demonstrated that simultaneous inhibition of both IKKs with a pharmacologic inhibitor rapidly induces apoptosis in the neonatal PEC.^8^ To determine the separate functions of IKKα and IKKβ on PEC survival, we measured caspase 3/7 activation in the NTC, IKKα and IKKβ siRNA‐treated cells. In response to serum withdrawal, apoptosis increased over time for all 3 groups (Figure 3A), with higher peaks at 8 and 24 hours compared to 4 hours (*P* < .0001 for NTC siRNA, IKKα siRNA and IKKβ siRNA‐treated cells). The IKKα depleted cells also demonstrated slightly increased apoptosis at 4 hours, and significantly higher levels at 24 hours as compared to NTC cells. In the IKKβ depleted cells, levels of apoptosis were similar to controls at 4 and 8 hours, but significantly increased by 24 hours compared to NTC or IKKα siRNA‐treated cells.

Stimulation with TNF‐α, a cytokine that induces apoptosis in PECs,^20^ more markedly increased apoptosis over time in all siRNA transfected groups (Figure 3B), with higher caspase 3/7 activation at 8 and 24 hours compared to 4 hours (*P* < .0001 for NTC siRNA, IKKα siRNA and IKKβ siRNA‐treated cells). Loss of IKKα worsened cell survival in response to TNF‐α at 8 hours, with over 50% greater levels of active caspase 3/7 as compared to NTC siRNA‐treated cells (*P* < .0001). However, by 24 hours, the levels of apoptosis became similar to controls. Loss of IKKβ caused a similar, but more sustained increase in apoptosis in response to TNF‐α compared to NTC‐cells at 8 hours, and significantly higher levels compared to both NTC and IKKα transfected cells at 24 hours.

### 3.4 Silencing IKKα and IKKβ induce distinct abnormalities in cell adhesion and motility

**Figure 4:**
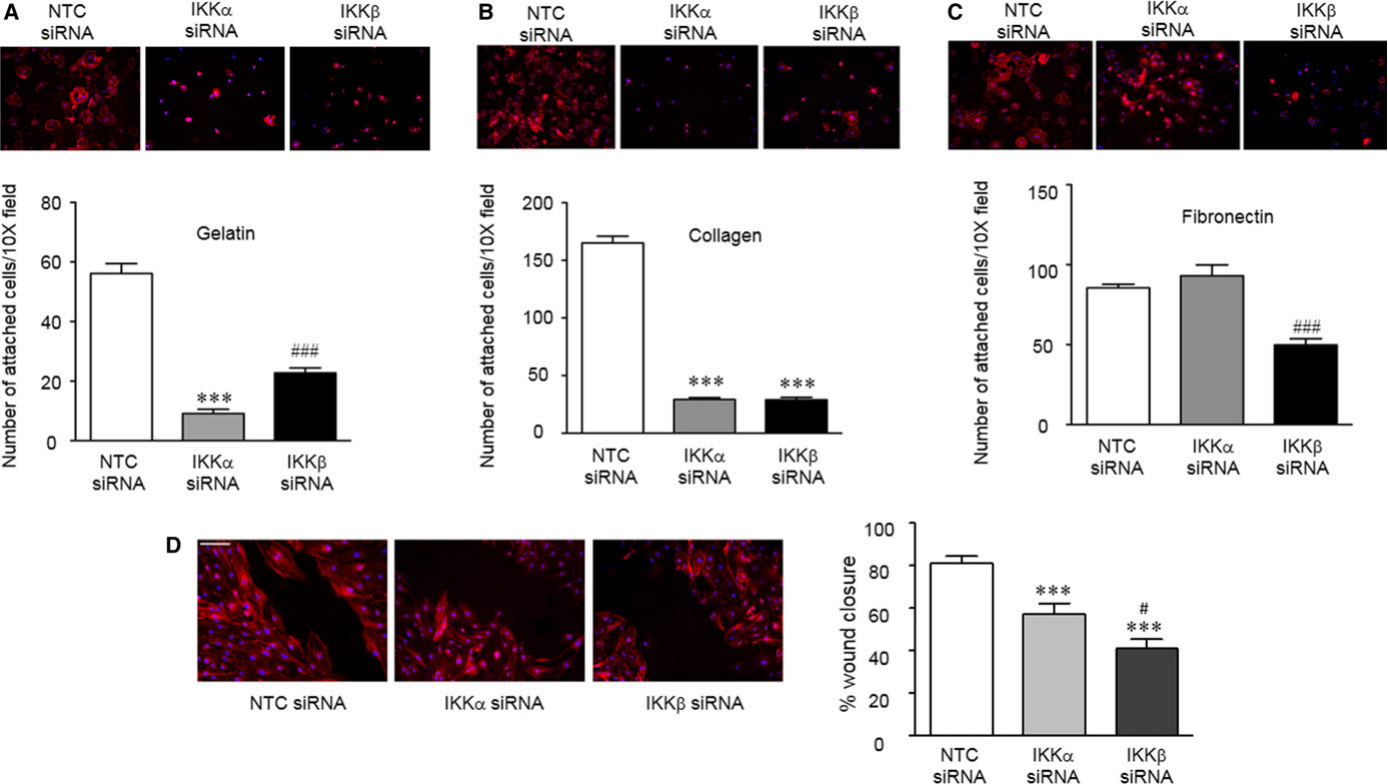
Silencing IKKα and IKKβ induce distinct abnormalities in cell adhesion and motility. Cell adhesion assays were performed with the NTC‐, IKKα‐ and IKKβ siRNA‐treated cells at 48 h after transfection. In each case, cells were seeded into wells coated with (A) gelatine, (B) collagen or (C) fibronectin, and after 1 h, non‐adherent cells were removed, and adherent cell stained and counted. Data shown are mean ± SEM with n = 9‐10. ***P < .001 vs NTC siRNA‐treated PEC, and ^###^ *P* < .001 vs IKKα siRNA‐treated PEC. Representative images of adherent cells stained with Phalloidine‐TRITC. (D) Wound healing assay performed with the NTC‐, IKKα‐ and IKKβ siRNA‐treated cells, and the percent area of wound closure calculated at 24 h. Data shown are mean ± SEM with n = 8. ***P < .001 vs NTC siRNA‐treated, and ^#^ *P* < .05 vs IKKα siRNA treated PEC

In addition to proliferation and survival, effective angiogenesis requires coordinated steps of cell motility, including the attachment of cellular protrusions to the extracellular matrix.^21^ Thus, we next evaluated the roles of IKKα and IKKβ on mediating cell adhesion to 3 matrices: gelatine, type II collagen and fibronectin. NTC‐cells adhered best to collagen (Figure 4B), and least effectively to gelatine (Figure 4A). Loss of IKKα disrupted cell adhesion to both collagen and gelatine, but adhesion to fibronectin (Figure 4C) was preserved. In contradistinction, loss of IKKβ impaired adhesion to all 3 matrices, although adhesion to gelatine was less affected than that observed after the loss of IKKα.

We also evaluated the impact of silencing IKKα, and IKKβ on PEC migration by wound‐healing assays in the NTC, IKKα and IKKβ siRNA‐treated neonatal PEC.^22^ NTC‐PECs at the leading edge of the wound demonstrated prominent central stress fibres, and rapidly organized perpendicular to the short axis of the wound (Figure 4D). In contrast, both the IKKα and IKKβ siRNA‐treated cells demonstrated decreased stress fibre formation and a random disorganization of polarity at the wound edge. Ratio of the wound closure at 24 hours demonstrated that although the loss of either IKKα or IKKβ impaired wound healing, loss of IKKβ had a more pronounced effect (Figure 4D).

### 3.5 Silencing IKKα and IKKβ causes distinct defects in actin cytoskeletal rearrangement

**Figure 5:**
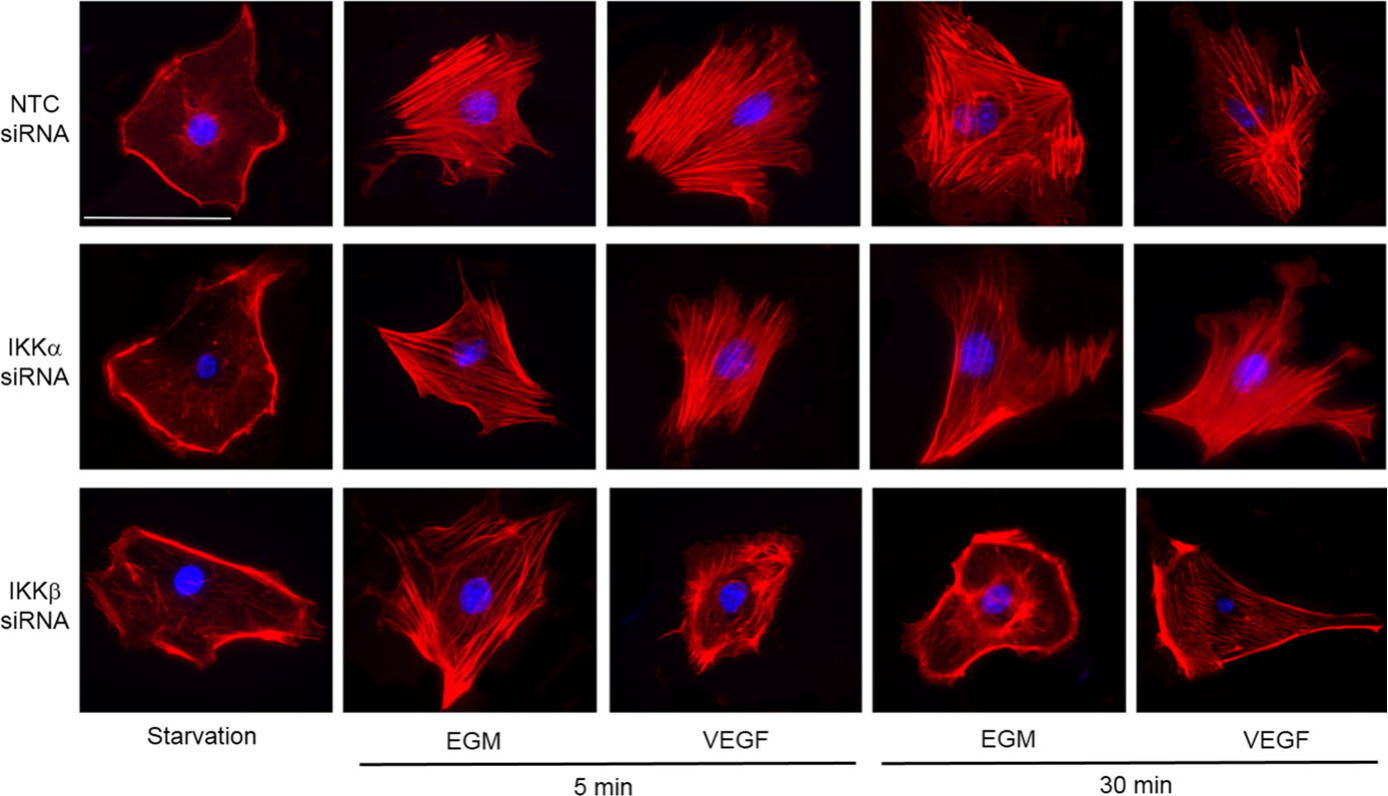
Silencing either IKKα or IKKβ causes distinct abnormalities in actin cytoskeletal rearrangement. Representative confocal images of NTC‐, IKKα‐ and IKKβ siRNA‐treated cells at 5 and 30 min after stimulation with starvation media (FBS 0.2%), complete EGM or starvation media containing VEGF (50 ng/mL). Calibration mark equals 100 μm

Cell migration requires coordinated changes in the remodelling of the actin cytoskeleton to form filopodia, lamellipodia and stress fibres.^21^ We next evaluated the effect of silencing either IKKα or IKKβ on cytoskeletal remodelling at baseline, and in response to growth factor stimulation. There were no obvious differences in the actin cytoskeleton in the NTC, IKKα and IKKβ siRNA‐treated PEC under short‐term starvation conditions of 12 hours (Figure 5). We chose to starve the cells for a short term to avoid any apoptosis implication in the process. In all 3 groups, cortical actin was prominent, with the absence of stress fibres. In the NTC‐cells, treatment with either EGM or VEGF induced prominent parallel stress fibres apparent by 5 minutes, and persistent stress fibres at 30 minutes. The IKKα siRNA‐treated cells also formed stress fibres in response to both EGM and VEGF at 5 minutes, although the fibres appeared to be slightly fewer in number. By 30 minutes, the stress fibres present in IKKα‐depleted cells were appreciably thinner than those observed in control cells. The IKKβ siRNA‐treated cells stimulated with EGM formed stress fibres that were thinner and less well‐aligned than those observed in the NTC‐ cells at 5 minutes, and VEGF‐stimulated cell demonstrated a marked abnormality of stress fibre formation, with disorganized actin primary localized at the periphery. At 30 minutes, stress fibre formation was markedly abnormal in both the EGM‐ and VEGF‐stimulated IKKβ‐depleted cells, showing prominent cortical actin and a paucity of stress fibre formation.

### 3.6 Effects of silencing IKKα and IKKβ on MAPK activation

**Figure 6:**
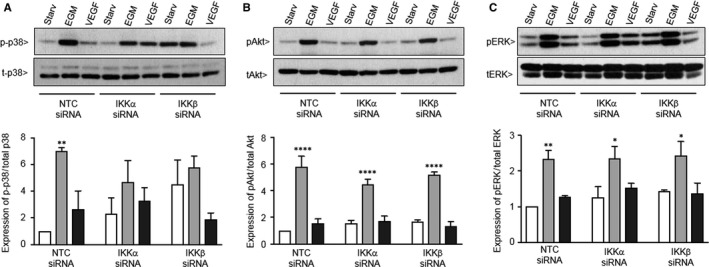
Silencing IKKα and IKKβ does not significantly alter MAP kinase activation after 5 min of growth factor stimulation. Representative Western blots to detect phosphorylated and total p38 (A), Akt (B) and ERK (C) 5 min after stimulation with starvation media (0.2% FBS), EGM, or starvation media containing VEGF (50 ng/mL). Data shown are the mean ± SEM of n = 3 immunoblots, with the amount of protein in each band expressed as the fold change over the NTC starvation sample. *P < .05, **P < .01 and ****P < .0001 vs starvation

Vascular endothelial growth factor‐mediated angiogenesis is associated with the activation of additional intracellular signalling pathways that promote angiogenic function, including activation of the MAPK signalling. Specifically, activation of ERK1/2 promotes endothelial cell proliferation, and activation of p38 promotes actin reorganization and cell migration.^23^, ^24^ Thus, we next determined the effect of silencing IKKα and IKKβ on MAPK activation. NTC‐cells demonstrated robust activation of p38 after stimulation with EC growth media, and a slightly lower activation of p38 in response to VEGF. Basal levels of active p38 trended to be higher in the IKKα and IKKβ siRNA‐treated cell, and they expressed a more modest activation in response to EGM that was not statistically higher than starvation (Figure 6A). However, mild abnormalities were not observed with the other MAP kinases interrogated, as both IKKα and IKKβ silenced cells demonstrated similar patterns of Akt and ERK1/2 activation as compared to control cells (Figure 6B,C).

### 3.7 IKKβ mediated regulation of VCAM serves to promote PEC adhesion and migration

**Figure 7:**
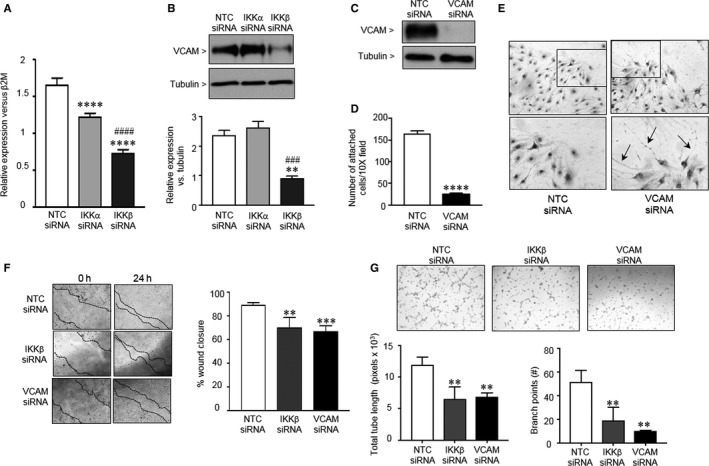
IKKβ mediated regulation of VCAM serves to promote PEC angiogenic function. (A) Quantitative PCR to detect VCAM gene expression relative to β2M in neonatal PEC treated with NTC, IKKα and IKKβ siRNA. Data shown are mean ± SEM with n = 12, and ****P < .0001 vs NTC siRNA treated, and ^####^ *P* < .0001 vs IKKα siRNA‐treated cells via one‐way ANOVA. (B) Representative Western blot to detect VCAM protein in neonatal PEC treated with NTC, IKKα and IKKβ siRNA. Data shown are mean ± SEM with n = 3, and **P < .01 vs NTC siRNA treated, and ^###^ *P* < .001 vs IKKα siRNA‐treated cells via one‐way ANOVA. (C) Representative Western blot to confirm effective knock‐down of VCAM protein in neonatal PEC treated with NTC and VCAM‐1 siRNA. (D) Cell adhesion assays were performed with the NTC‐ and VCAM siRNA treated cells at 48 h after transfection. Cells were seeded into wells coated with collagen I and after 1 h, non‐adherent cells were removed, and adherent cell stained and counted. Data shown are mean ± SEM with n = 10. (E) Representative phase contrast images of NTC and VCAM siRNA‐treated neonatal PEC at the migrating edge of a wound. Arrows indicate long, thin, abnormal filopodia extending from VCAM siRNA‐treated cells. (F) Wound healing assay performed with the NTC‐ and VCAM or IKKβ siRNA‐treated cells, and the per cent area of wound closure calculated at 24 h. Data shown are mean SEM with n = 15 **P < .01 and ***P < .001 vs NTC siRNA‐treated via one‐way ANOVA. (G) Tube forming assay performed with the NTC, VCAM or IKKβ siRNA‐treated cells. Left graph shows the total tube length for each treatment, and the right graph shows the number of branch points. Data shown are mean SEM with n = 3, with **P < .01 vs NTC siRNA‐treated cells via one‐way ANOVA

We next sought to identify downstream effector molecules regulated by the NFκB pathway that were promoting angiogenesis in the PEC. Given that silencing of either IKKα or IKKβ induced either mild or severe impairments in angiogenic functions, we focused on the small group of shared genes dysregulated by silencing either IKKα or IKKβ, such as vascular cell adhesion molecule (VCAM). Microarray analysis found VCAM to be down‐regulated 1.9‐fold in both the IKKα and IKKβ silenced cells. qPCR to validate these results identified a small, but statistically significant decrease in VCAM gene expression in the IKKα‐silenced cells, and a more marked decrease in the IKKβ‐silenced cells (Figure 7A). Despite the decrease in VCAM gene expression in the IKKα‐silenced cells, Western blot analysis did not reveal differences in VCAM protein levels between NTC and IKKα depleted PECs (Figure 7B). In contrast, VCAM protein was decreased by approximately 60% in the IKKβ siRNA treated PEC.

To confirm the importance of VCAM in modifying angiogenic function in PECs, we silenced VCAM expression using siRNA and assessed PEC adhesion, migration and in vitro tube formation. By Western blot, we confirmed almost complete loss of VCAM protein in the VCAM siRNA‐treated PEC (Figure 7C). Loss of VCAM markedly impaired PEC adhesion to collagen I (Figure 7D), to a degree similar to that which we observed after silencing of IKKβ (Figure 4B). Adhesion and migration defects were associated with phenotypic abnormalities of the PEC during wound closure (Figure 7E). NTC siRNA‐treated PEC located at the leading edge of the wound were organized in parallel, with some cells extending lamellipodia towards the direction of the scratch. In contrast, the VCAM siRNA‐treated PEC at the wound edge were disorganized, with many cells extending abnormally long, thin filopodia in multiple directions (Figure 7E). To qualify these defects in migration, we compared the effects of silencing VCAM with those induced by silencing IKKβ (Figure 7F) in a wound healing assay. Although the NTC siRNA‐treated PEC were able to effectively close the wound in the monolayer, similar impairments in wound healing were observed in response to silencing both IKKβ and VCAM. Similarly, using a method to assess VEGF‐mediated tube formation, we found that both IKKβ and VCAM siRNA‐treated PEC developed an overall decrease in the total length of tube formation, and a decreased number of branch points as compared to the NTC siRNA‐treated PEC (Figure 7G).

## 4 DISCUSSION

We previously identified the NFκB as an essential regulator of pulmonary angiogenesis during postnatal lung growth and alveolarization.^8^, ^13^ Here we report independent functions for IKKα and IKKβ in the developing pulmonary circulation, with IKKβ as the predominant regulator of angiogenesis during alveolarization.

In the canonical pathway of NFκB activation, IKKα and IKKβ work in concert to release NFκB dimers from the inhibitory IκBs, and allow their translocation to the nucleus to regulate gene expression. Our prior studies found that NFκB is endogenously active in neonatal PECs during early alveolarization, but the mechanism allowing for this developmental activation is not yet clear. Constitutive activity of NFκB is normally restricted to cells from the hematopoietic lineage.^25^ In other cell types, basal NFκB activity is negligible and induced only after stimulation.^26^ However, high levels of constitutively active NFkB are found in cancer cells, induced by paracrine secretion of cytokines and other factors from the tumour.^27^ Therefore, it is possible that secreted factors present in the lung microenvironment allow for temporal‐specific activation of the IKKβ/NFκB pathway in the developing pulmonary endothelium.

Despite significant sequence homology, silencing IKKα and IKKβ in PEC resulted in the dysregulation of both shared and unique genes. Both IKKα and IKKβ can exert distinct NFκB‐dependent and independent effects that vary in a cell stimulus‐specific manner.^5^ Their separate functions, and limited ability to compensate for each other are highlighted by the distinct embryonic phenotypes resulting from deletion of either IKKα or IKKβ.^28^, ^29^ Importantly, the IKK complex lacking IKKα is still able to phosphorylate IκB in vitro, suggesting that IKKβ is the primary activator of inducible NFκB activation.^30^ In addition to NFκB‐dependent effects, the IKKs can act differently on transcription factors,^31^, ^32^ signalling pathways,^33^ mRNA and protein stability,^34^, ^35^ chromatin structure.^36^ Further studies may determine if NFκB‐dependent mechanisms are responsible for inducing the IKKα and IKKβ shared targets, and conversely, if NFκB‐independent mechanisms are responsible for regulating the IKKα and IKKβ unique targets in the developing pulmonary endothelium.

These data add to the work of others associating the IKK/NFκB pathway with physiologic and pathologic angiogenesis. Tie2‐mediated −/− IKKβ phenotype of hematologic and endothelial cells disrupts liver and placental vascular development inducing embryonic lethality, while the ± deletion impairs post‐ischaemia neovascularization in adult mice.^11^ IKKα is overexpressed in the vasculature of lung adenocarcinoma^10^ and increased IKKβ has been noted in multiple tumour types.^37^ In both cancer and wound healing, the pro‐angiogenic effects of the IKK/NFκB pathway have primarily been attributed to the transcriptional regulation of pro‐angiogenic cytokines and growth factors.^6^, ^38^ In contrast, in both here and in our prior study, we show that IKKα and IKKβ have direct effects on PECs, controlling angiogenesis.

In our study, silencing either IKKα or IKKβ impaired PEC survival. Anti‐apoptotic functions of the IKK/NFκB pathway have been well described. Deletions of either IKKβ or the NFκB subunit RelA result in early embryonic lethality as a result of extensive liver apoptosis.^28^ In macrophages, loss of IKKα compromises survival by decreasing Akt phosphorylation.^39^ Numerous anti‐apoptotic genes are under transcriptional control of the NFκB pathway, including BCL‐2 families.^40^ NFκB also regulates apoptosis by influencing p53 stability.^41^ IKKα too has a separate anti‐apoptotic function, influencing the ability of CBP to bind to either p53 or RelA, thus regulating the balance between pro‐survival, NFκB signalling and pro‐apoptotic p53 signalling.^41^, ^42^

We found that IKKβ was clearly the predominant regulator of angiogenesis with more profound defects in proliferation, adhesion, migration and cytoskeletal remodelling. In the IKKβ depleted cells, proliferation was impaired in response to EGM, VEGF and FGF‐2. Both VEGF and FGF‐2 are important growth factors that regulate angiogenesis during development and disease.^43^, ^44^ Interestingly, the IKKβ depleted cells demonstrated a non‐significant trend in increased cell proliferation in response to IGF‐1, IGF‐1 is expressed in blood vessels of neonatal but not adult animals, and expression is increased in budding capillaries.^45^ Blocking the IGF‐1 receptor in human and rat foetal lung explants induces endothelial cell loss, increases mesenchymal cell apoptosis and disrupts lung development.^46^ This suggests that in the absence of effective VEGF or FGF‐2 signalling, compensatory mechanisms through IGF axis may be invoked in the IKKβ depleted cells to preserve proliferation.

Vascular endothelial growth factor signalling through the VEGFR2 receptor is an essential pathway mediating postnatal pulmonary angiogenesis during alveolarization.^47^, ^48^ VEGF is decreased in the lungs of premature infants dying from BPD and in the lungs of animal models of disrupted alveolarization and pulmonary angiogenesis.^3^, ^49^, ^50^, ^51^ Blocking VEGF/VEGFR2 signalling in neonatal rats decreases pulmonary arterial density and impairs alveolarization,^52^ while overexpression of VEGF in newborn rats exposed to hyperoxia preserves pulmonary angiogenesis and alveolarization and increases survival.^53^ Loss of IKKβ appeared to result in specific defects in VEGF‐mediated signalling within the IKKβ depleted cells stimulated with VEGF demonstrating severe alterations in actin remodelling, including the absence of stress fibres and increased cortical actin at the periphery. These cytoskeletal abnormalities were still apparent, but milder in the IKKβ depleted cells stimulated with EGM, suggesting that additional growth factors in the media may partially compensate for this defect in VEGF signalling. Despite these defects, activation of MAP kinases which promote cell migration, such as p38 were only mildly impaired, suggesting that other effector molecules may be primarily responsible.

We further identified VCAM as one IKKβ regulated target that appears to play a significant role in promoting normal PEC adhesion, motility and in vitro angiogenesis (Figure 7F,G). VCAM‐1 is expressed in response to inflammatory cytokines and growth factors and promotes the adherence of leucocytes to activated endothelial cells.^54^ However, additional roles for VCAM‐1 in embryonic development and pathologic neovascularization have recently been described. Deletion of VCAM‐1 induces embryonic lethality between E10.5 and E12.5, resulting in failure of fusion of the allantois to the chorion, and of the endocardium to the myocardium with subsequent cardiac haemorrhage.^55^ Moreover, a soluble form of VCAM has also been shown to promote angiogenesis, serving as a chemotactic agent for endothelial cells in inflammatory diseases.^56^ To our knowledge, this is the first report suggesting a role for VCAM in promoting physiologic angiogenesis during development. Silencing of VCAM markedly disrupted PEC adhesion and migration, and induced the appearance of abnormal filopodia formation. Endothelial filopodia serve as antennae, detecting chemical and mechanical cues in the environment. Motility is directed by anchoring filopodial protrusions and subsequent retraction of the cell body, moving the cell forward.^57^ Further studies will need to explore whether the effect we observed in the VCAM depleted cells is secondary to decreases in soluble VCAM expression, or if cell surface VCAM has a previously unrecognized function in mediating appropriate sensing and anchoring of filopodia.

In addition to VCAM, we found that IKKα and IKKβ regulate over 700 genes in the PEC. Although the scope of this study did not permit a comprehensive investigation of all the putative downstream targets that may have a role in mediating angiogenic function in the PEC, there were numerous candidates identified by microarray that could play critical roles. These include targets that are important in regulating discrete steps in cell motility such as Rac3,^58^ Rho GTPases (PAK1)^59^ and antiangiogenic factors (IGFB5 and FLT‐1).^17^, ^60^ Further, silencing of IKKα dysregulated more genes than silencing of IKKβ, yet the effect of IKKα depletion on PEC angiogenic function was relatively modest. These data suggest that IKKα may be an important regulator of non‐angiongenic homoeostatic functions in pulmonary endothelial cells that may warrant further investigation.

There are a number of limitations to our study. First, given that the loss of both IKKα and IKKβ also induced impairments in cell survival it is impossible to fully separate the effects of cell death on other measures of angiogic function, including adhesion, migration and in vitro angiogenesis. However, the greater degree of cell death was observed in response to TNF‐α stimulation, or in response to prolonged serum starvation. In attempt to limit these confounders as much as possible, we performed these function assays with either no or short periods of starvation, and with assay times that were also short in nature. Second, although our prior work demonstrated that pharmacologic inhibition of both IKKα and IKKβ effectively impairs pulmonary angiogenesis and disrupts alveolarizartion in neonatal mice in vivo,^8^ definitive evidence for a role for IKKβ in promoting pulmonary angiogenesis in vivo using an inducible, cell‐specific model allowing EC‐specific deletion at the start of alveolarization has not been done.

In summary, we explored the specific roles of IKKα and IKKβ in the developing pulmonary endothelium. Silencing either IKKα and IKKβ disrupted hundreds of genes, but the panels of genes regulated by the 2 kinases were distinct, suggesting that each kinase has separate functions in the pulmonary endothelium of developing vessels. Additional studies to assess the functional roles of IKKα and IKKβ in the pulmonary endothelium demonstrated that IKKβ plays a predominant role in promoting a broad array of cellular functions including survival, proliferation, adhesion, migration and actin reorganization. These angiogenic defects were more exaggerated in cells stimulated with VEGF alone, suggesting a specific impairment in VEGF‐mediated angiogenesis. VCAM was identified as an IKKβ‐regulated downstream target, and silencing of VCAM alone resulted in significant impairments in cell adhesion, migration and tube formation, identifying an important, but previously undescribed role for VCAM in pulmonary vascular development. Angiogenesis is essential for alveolarization, the stage of lung development that exponentially increases gas exchange surface area. Disrupted angiogenesis contributes to many paediatric diseases, including bronchopulmonary dysplasia, and therapeutic strategies to enhance angiogenesis may have potential benefit to these and other lung diseases marked by impaired angiogenesis. Our prior study showed that disrupting IKK‐mediated signalling disrupts both angiogenesis and alveolarization. Data from this study identify IKKβ as the predominant regulator of angiogenic function in developing pulmonary endothelial cells and suggest that strategies aimed to specifically preserve and enhance IKKβ activity may be an important therapeutic strategy to preserve late lung growth.

## CONFLICT OF INTEREST

The authors confirm that there are no conflicts of interest.
